# Physical Practice and Wellness Courses Reduce Distress and Improve Wellbeing in Police Officers

**DOI:** 10.3390/ijerph15040578

**Published:** 2018-03-23

**Authors:** Daniela Acquadro Maran, Massimo Zedda, Antonella Varetto

**Affiliations:** 1 Department of Psychology, Università di Torino, Via Verdi 10, 10124 Torino, Italy; massimo@zedda.it; 2 Città della Salute e della Scienza, Corso Bramante, 88, 10126 Torino, Italy; avaretto@cittadellasalute.to.it

**Keywords:** work-related stress, coping strategies, mental health, police

## Abstract

Background: The aim of this work was to evaluate a course to reduce distress in an Italian police force. Based on the findings from the first investigations on this population, courses to improve the ability to manage distress were tailored by management. Several free courses were proposed, including physical efficiency (e.g., total body conditioning) and wellness (e.g., autogenic training) classes. The goal of this research was to evaluate the courses and their impact on the perceived distress and general health of the participants, as well as the effectiveness in increasing the use of adaptive coping strategies. Methods: A descriptive investigation was conducted involving a sample of 105 police officers before (time 1) and after (time 2) they had participated in the courses. Results: Findings confirmed both physical and wellness courses affected, in participants, the perceived distress, thereby increasing the perception of wellbeing. The participants expressed having mental health benefits, the use of adaptive coping strategies increased, while the maladaptive coping strategies decreased. Conclusion: This study confirms that these courses could effectively reduce the risk of chronic disease, a consequence of persistent exposure to distress.

## 1. Introduction

There is a growing body of literature investigating work-related distress in police officers, a profession that is understood to be dangerous and stressful [1,2]. The sources of distress and the strategies to cope with them, as well as the consequences, in terms of individual health and organizational wellness, have been well described. The sources of distress are due to the nature of the job, including both operational (e.g., patrol activities, community services, crime prevention, criminal investigations, and traffic control) and organizational tasks (e.g., bureaucratic procedures, record keeping, and public information) [3,4]. Most of these tasks are typical, and thus, some work organization interventions (e.g., schedules, shift distribution) could be repurposed as other interventions that are inherent to the job (e.g., road accident interventions) [5]. The consequences of distress affect the health of this population; police officers suffer from physical disorders, such as sleep disorders [6], cardiovascular diseases [7], and stomach troubles, as well as emotive troubles, such as anxiety, depression, flashbacks, and panic attacks [8,9]. These consequences affect the perception of an unsafe working environment. This may predispose police offers to the generalized unsafety theory of stress [10] that identifies and explains far more stress-related physiological activity that is responsible for chronic diseases, such as asthma [11], coronary artery disease [12], and obesity [13]. Those consequences could also affect quality of life, with behavioral outcomes, such as marital problems, substance use, and social avoidance [14]. Consequently, organizations suffer from worker absenteeism, due to stress-related injuries, reduced productivity, and costs associated with turnover [15]. To cope with distress, police officers use different strategies. The literature results have shown that police officers use both maladaptive (such as alcohol misuse) and adaptive (such as humor) coping strategies, which have had different impacts on individual wellbeing; the use of avoidant strategies causes a higher level of distress, which could become chronic [16]. To improve the ability to manage distress, scholars have purposed various programs to promote health, wellness, and safety. Those programs assume a variety of forms and focus on fitness, nutrition, weight management, musculoskeletal conditioning, resilience to trauma, and so on. As reported by Kuhns, Maguire, and Leach [17], most programs focus on improving physical condition (working with police officers on fitness levels, diet, and exercise practices) and wellness (working on the ability to manage negative emotion through, for example, meditation). The findings from investigations on the impact of physical and wellness programs have shown a decrease in the level of perceived distress, and increases in the level of positive feelings, the ability to cope with stressful situations, and mental health benefits [18,19,20]. 

The aim of this work was to evaluate a course to reduce distress in an Italian police force. The originality of this research project was that the request for a course was made at the end of a previous investigation involving a population of municipal police officers operating in a region in the northwest portion of Italy. The findings from the first investigations [21,22] showed a high risk of distress in patrol officers, both women and men, in all roles and sectors of the intervention. The consequences affected their wellbeing; this population suffered from emotional problems (e.g., sadness) and some forms of somatization (e.g., dry/congested nose). Distress also caused behavioral problems. The police officers involved in the investigation described practical (e.g., childcare) and family problems (e.g., “dealing with partner”). Moreover, the overall findings showed that the study population generally demonstrated the use of both maladaptive and coping strategies, confirming a worse state of health in those respondents who were more prone to using maladaptive coping strategies, such as behavioral disengagement and self-blame. Based on these findings and replies to open questions [21], the management tailored a course to improve the ability to manage distress. Several free courses were proposed, including physical efficiency (e.g., total body conditioning) and wellness (e.g., autogenic training) classes. All courses were held outside working hours at a gym located far from the workplace. However, more patrol officers enrolled than expected, therefore, the same course was held multiple times during the year, confirming the need of this population to manage distress and improve their wellbeing. The goal of this research was to evaluate the courses and their impact on the perceived distress and general health of the participants, as well as the effectiveness in increasing the use of adaptive coping strategies. A descriptive investigation was conducted involving a sample of police officers before (time 1) and after (time 2) they had participated in the courses. 

## 2. Method

In September 2016 (time 1) and in September 2017 (time 2), a survey was conducted to measure the distress and wellbeing experienced by the patrol officers from the physical practice and wellness courses, as well as the coping strategies that they used. The quantitative method was chosen because it permits an assessment of the effects of exposure to distress, the consequences, and how participants respond to these courses. The police officers could choose to participate in one of the following courses: postural gymnastic, tai chi chuan, total body conditioning, autogenic training, yoga, or dynamic meditation. The courses were categorized based on their focus (physical practice: postural gymnastic, tai chi chuan, total body conditioning; wellbeing: autogenic training, yoga, dynamic meditation). The data were analyzed to obtain the means and to determine the effect of the course on the participants confronting the variables before and after participating in the courses. The research can be replicated, due to its ability to compare the variables in time 1 and time 2 through the use of closed-end questions, structured approaches, and statistical procedures [23].

### 2.1. Ethical Statement

The study conformed to the provisions of the Declaration of Helsinki in 1995, revised in Edinburgh 2000 (World Medical Association, 2001) [24]. All ethical guidelines were followed, as required for conducting human research, including adherence to the legal requirements of Italy (the latest concerning the statement about privacy). The research project was approved by a committee composed of two unit managers (one with a degree in law, the other a clinical psychologist) and one supervisor (the head of general affairs). As there was no medical treatment or other procedures that could cause psychological or social discomfort to participants, additional ethical approval was not required. With the approval of the managers, the participants were asked for authorization to administer the questionnaires in time 1 and time 2. Each participant made a personal code (nickname) that permitted their identification in the two questionnaires (time 1 and time 2). The personal codes were unknown to the researchers, and they were deleted at time 2; the personal code was replaced with a number (001, 002, and so on). The cover sheet clearly explained the research aim, the voluntary nature of the participation, the anonymity of the data, and the elaboration of the findings. Thus, returning the questionnaires implied consent. The participants volunteered for the research without receiving any reward.

### 2.2. Participants

A total of 105 police officers, including 36 (34.3%) men and 69 (65.7%) women, participated in the study. The average age was 48.23 years (SD = 5.98, range 26–60 years). Most participants were married (47, 44.8%), 24 (22.8%) were separated, 19 (18.1%) were single, 14 (13.4%) were cohabiting/engaged, and one was a widower. Most respondents (70, 66.6%) had one or more children. Less than half (49, 46.7%) of the respondents participated in one of the physical practice courses, and most were female (33, 67.3%). The average participant age was 47.4 years (range 26–60; SD = 5.71). Most were married (27, 48.2%), and 40 (71.4%) had one or more children. More than half (56, 53.3%) participated in one of the wellness courses, and most were female (33, 67.3%). The average age was 49.2 years (range 34–59; SD = 6.20). Most were married (20, 40.8%), and 30 (61.2%) had one or more children.

### 2.3. Materials

The self-administered questionnaires in time 1 (pre-participation) and time 2 (post-participation) were composed of the same sections. In time 1 and time 2, the first part of the questionnaire contained the introduction and the purpose of the research, the instructions for completing the questionnaire, and the anonymity and privacy statements, followed by the Goal Attainment Scale, the General Health Questionnaire, the Distress Thermometer and the Brief COPE. 

*Goal Attainment Scale (GAS)*. This scale was used to define individual realistic and feasible goals, according to the participants’ needs and expectations. In this study, GAS [25,26,27] was measured using a 5-point Likert scale ranging from −2 (no change) to +2 (overachievement). In time 1, the request was to indicate the principal goal for participating in the course (only one answer, e.g., “to decrease the stress experienced with citizens and/or with colleagues”). In time 2, this section was replaced with a request to indicate how the goal was achieved. The possible answers were as follows: −2 (worse expected outcome), −1 (less than expected outcome), 0 (expected outcome), +1 (more than expected outcome), and +2 (best expected outcome) (Cronbach’s alpha = 0.66). 

*General Health Questionnaire-12 (GHQ-12).* The scale was used to evaluate the participants’ mental health over the previous few weeks. The scale is a 12 item screening instrument used to detect psychological distress [28] (e.g., “over the past few weeks, have you been able to concentrate on whatever you are doing?”). Responses were given based on a four-point Likert scale (0–3) ranging from “better than usual” to “much less than usual” [29]. The scoring method (0-0-1-1) was used, as suggested by the authors [28]. The scores ranged from 0 to 36, with higher scores indicating greater distress. This study had a Cronbach’s alpha coefficient of 0.69.

*Distress Thermometer (DT)*. Using this rapid screening instrument, the respondents rated their perceived level of distress during the previous week on a visual analogue scale that ranged from 0 (not distressed) to 10 (extremely distressed) [30]. Most studies use a cut-off score of 4. The DT also investigated the sources of distress: practical problems (5 items, e.g., “childcare”), family problems (3 items, e.g., “dealing with partner”), emotional problems (6 items, e.g., “sadness”), spiritual/religious concerns (1 item), and physical problems (21 items, e.g., “dry/congested nose”). In this study, the Cronbach’s alpha was 0.70. 

*Brief COPE*. This was used to assess the following coping strategies: self-distraction, active coping, denial, substance use, emotional support, instrumental support, behavioral disengagement, venting, positive reframing, planning, humor, acceptance, religion, and self-blame [31]. This scale rates 28 coping responses under stressful conditions. The scores range from 1 (I would not normally do this) to 4 (I would usually do this). In this study, the Italian version was used [32] (Cronbach’s alpha = 0.67).

Statistical analyses were performed using SPSS statistical software (version 24, SPSS Inc., Chicago, IL, USA). Descriptive measures (means ± SD) were calculated for all test variables for all groups of participants. A *t* test for paired samples was performed to evaluate the statistical significance of the observed differences (time 1, time 2). One-way ANOVA, with the Bonferroni post hoc test, was used to assess the differences between the mean perceived mental health and distress scores, the sources of distress, and the coping strategy scores for achievement of the goals expressed by the participants, and *p* < 0.05 was considered to be statistically significant.

### 2.4. Procedure

The time 1 version of the questionnaire was distributed to 105 police officers ten days before the beginning of the course, including the participants in the wellness course and those participants in the physical practice course. All courses started on October 2016 and ended in May 2017; thus, the distribution at time 1 was performed the last week of September 2016. Each course had a weekly activity, and the activity duration was 1.5 h. The distribution to the participants was performed by the manager of the courses, who presented the aim of the study. The participants were asked to complete all parts of the questionnaire and place it into a sealed box that had a slot in the top, which was left in their changing rooms. This procedure was adopted from previous studies [21,22] and enabled us to guarantee participant privacy and anonymity. On the box, there was a label indicating the title of the study, the expected collection date, and the contact details of one of the authors of this paper, for anyone who required more information. None of the participants used this opportunity. The questionnaires were collected seven days later. The same procedure was used for time 2. The questionnaires were collected three months after the end of the courses, during the first week of September 2017.

## 3. Results

In time 1, the participants indicated the goal of the participating in the courses. Most of the participants in the physical practice course (28, 57.1%) indicated “to increase well-being”, while 21 (42.9%) indicated “to decrease the distress experienced with colleagues and/or citizens”. For time 2, the results showed that most participants achieved the goal; only six (12.2%) indicated that the goal was achieved in a “less than expected” manner. Similarly, most (36, 64.3%) participants in the wellness courses indicated that their goal was “to increase wellbeing”, and 20 (35.7%) indicated that the goal was “to decrease the distress experienced with colleagues and/or citizens”. Moreover, participants indicated overall satisfaction with the goal by participating in the courses, only two (3.6%) indicated that the goal was achieved in a “less than expected” manner. In both groups, none of the participants indicated “worse than expected”. Overall, the ANOVA results showed that the scores significantly affected positive reframing (*F* = 5.93, *p* = 0.001) and acceptance (*F* = 5.30, *p* = 0.003) coping strategies. Bonferroni post hoc analyses showed that the participants who indicated a “best expected” goal indicated a positive reframing coping strategy more frequently than the participants who indicated “expected outcome” (*p* = 0.019) and “more than expected outcome” (*p* = 0.001). For the acceptance coping strategy, the Bonferroni post hoc analysis showed that the participants who indicated a “best expected” goal indicated this strategy more than the participants who indicated “less than expected outcome” (*p* = 0.035), “expected outcome” (*p* = 0.003), and “more than expected outcome” (*p* = 0.002). 

The GHQ-12 findings showed that for the participants in the physical practice and wellness courses, the perception of wellbeing increased: the scores were lower in time 2 than in time 1 (see Table 1 and Table 2). The same trend emerged for the DT scores. The participants indicated less perceived distress in time 2 than in time 1. There were some differences in the problems describing the source of the distress. The participants in the physical practice course indicated fewer problems (practical, emotional, and physical) in time 2 than in time 1, while the participants in the wellness courses indicated only fewer emotional problems in time 2 than in time 1. The coping strategies trend was similar: the number of adaptive coping strategies increased (i.e., acceptance, religion, and positive reframing—the latter only in participants in the physical practice course), except for emotive support, which decreased. Maladaptive coping strategies tended to decrease (self-blame, negation, substance use, behavioral disengagement). Interestingly, self-distraction and active coping received different scores from the participants in the physical practice and wellness courses. The participants in the physical courses indicated less self-distraction and more active coping in time 2 than in time 1. Participants in the wellness course, on the contrary, indicated more self-distraction and less active coping in time 2 than in time 1. 

## 4. Discussion

Our study findings confirmed that both physical and wellness courses affected the perceived distress [20], thereby increasing the perception of wellbeing [18,19]. The participants in the physical and wellness courses expressed having mental health benefits, and the DT and GHQ scores significantly decreased in time 2. Moreover, the physical practice participants indicated that they improved their ability to address some problems, which could suggest better management of the time devoted to participating in the courses and work commitments, while the family commitment remained a stable problem over time in both groups of participants. The participation in physical practice and wellness courses also affected the ability to cope with stressful situations [17,20]. Overall, the use of adaptive coping strategies increased, while the maladaptive coping strategies decreased. When an individual began to develop adaptive coping strategies, he/she created a virtuous circle: the positive feeling associated with the use of adaptive strategies to cope with stressful situations helped to maintain the same strategies [33,34]. To create this circle, it is necessary to make sense of the experience, and to reflect upon and have insight into what one is feeling [35]. Without this process, any behavioral technique is experienced as a physical exercise. The behavioral techniques, particularly those based on life philosophies (e.g., yoga and dynamic meditation) imply a greater awareness of the physicality and mind–body connection [36]. This connection permits the participants to better face the sources of their distress (e.g., relationships with colleagues/citizens); thus, this positive feeling is the motive to choose to participate and continue the participation in the courses, even if they are organized outside working hours and in a gym far from the workplace. One interesting finding related to the GAS scores was that the acceptance coping strategy score increased in the participants who showed overachievement of the goal. The increased acceptance score indicates a psychological state that is relatively stable, and that permits a cognitive reappraisal that facilitates problem solving [37]. This implies that the participants identified some aspects of their personality, recognized it as having important qualities, and were able to use mental and behavioral activities that activated positive self-care or rational self-talk to accept their work experiences [38]. In terms of the positive reframing coping strategy, the increased score suggests that the participants reinterpreted their roles as police officers in a more positive manner, thereby perceiving a positive aspect of a situation that is traditionally viewed negatively [39]. Besides psychological benefits, the participation in physical practice and wellness course may offer biological benefits. Exposure to job-related stressors may confer increased risk for posttraumatic stress disorder (PTSD) among police officers [40,41,42]. The physical practice and wellness course may be able to reverse biological changes due to stress, and further research is required.

This study had several limitations. The lack of a control group attenuated our ability to draw definitive conclusions regarding the impact of physical practice and wellness courses on changes in perceived distress, increased wellbeing, and adaptive coping strategies in police officers. Moreover, other changes (e.g., organizational, occupational) during the investigation were not monitored in this population. Furthermore, in time 2, the questionnaire was administered on September 2017, the first week. In some cases, the participants may have recently returned from vacation; thus, the decrease in the scores of perceived distress and the increase in wellbeing might be linked to the vacation. Another limit is that we did not consider some variables, such as gender and sense of belonging to the group. Most participants were female (65.7%), while half of the police officers in this organization were male. Traditionally, females are more prone to search for constructive coping strategies to reduce distress and its consequences, including suicide ideation [43,44,45,46,47,48]. 

Study of this issue could benefit from the involvement of a larger sample of police officers participating in the physical practice and wellness courses, as well as a longitudinal study. We did not investigate the sense of belonging to a group: the decrease in the level of distress and the increased wellbeing could be linked with the feeling of being part of a group [49], with the opportunity to share negative emotions and find new solutions to handle stressful situations. The sense of belonging in this population needs to be explored in future research. In addition, it would be interesting to use a mixed approach (quantitative and qualitative, e.g., questionnaires and focus groups). This could be a useful source of data, evoking personal experience, capturing interpersonal dynamics, discussing sensitive topics. Moreover, psychological stress is associated with poor work attendance [50], work-related disability, and productivity loss [51]: further research is required to assess potential benefits of the courses on the above outcomes.

## 5. Conclusions

Despite the limits described above, this study confirms that physical practice and wellness courses decreased the perceived distress and increased the wellbeing in police officers. Consequently, these courses could effectively reduce the risk of chronic disease, a consequence of persistent exposure to distress. The six specific courses were chosen based on the need expressed by this population [21,22], thereby confirming that the findings from these investigations were well accepted by police management. Police officers benefited from participating in the courses; thus, the suggestion is to continue to propose these courses to all police officers. A better explanation of the benefits of participating in the courses should be communicated to all police officers, the hope is that a great number of police officers participate. Furthermore, not all police officers recognize their needs, and the risk is not to consider the mind–body connection as a possible solution to distress and its consequences. Psychoeducation should be offered to explain the relationship between stress and negative effect on hormonal and immunological responses which can lead to chronic diseases (e.g., cancer, heart diseases, and cognitive impairment) [52].

In particular, male police officers could have more difficulty expressing the negative feeling associated with distressful situations, due to the high male-norm pressure. As argued by Habersaat and colleagues [53], work-related stress may be expressed in other environments, such as the home, where normative pressure is lower [54]. The constant detection of organizational and occupational distress could help to monitor the consequences of work-related stress in police officers, paying attention to the situations at risk of maladaptive strategies (e.g., substance misuse) that could lead to a progressive deterioration of quality of life. For example, police officers could use smartphone alcohol tracker applications to monitor alcohol consumption, and alerts should be sent to police officers to avoid excessive drinking [55]. Moreover, prompt intervention in police officers who are facing stressful situations (e.g., road accidents or domestic violence) could be helpful to manage not only individual instant distress [56], but also to provide the opportunity to reflect on the need to improve one’s own ability to cope with it. Our hope is that this work could contribute to a better understanding of the importance of the physical practice and wellness courses for police officers. These courses could improve the quality of life in police officers, decreasing their distress levels and increasing their wellbeing. 

## Figures and Tables

**Table 1:** Means, Standard Deviation, Standard Error and Confidence Interval for Scores on the GHQ-12, Distress Thermometer and Brief COPE. Participants in physical practice courses.

Measure	Time 1		Time 2		95% Cl (LL–UL)	*t*	*p*
	M (SD)	SE	M (SD)	SE			
GHQ-12	5.23 (2.01)	0.27	3.45 (1.57)	0.21	1.16–2.41	5.77	0.000
DT:	4.62 (2.24)	0.36	2.79 (2.04)	0.33	0.93–2.71	4.15	0.000
Practical Problems	1.05 (1.10)	0.14	0.66 (0.86)	0.11	0.04–0.75	2.23	0.030
Family Problems	0.86 (0.84)	0.11	0.66 (0.72)	0.10	−0.11–0.50	1.30	0.201
Emotional Problems	1.82 (1.22)	0.16	1.36 (0.88)	0.12	0.07–0.86	2.33	0.023
Spiritual/religious concerns	0.04 (0.19)	0.03	0.07 (0.26)	0.04	−0.12–0.05	−0.81	0.419
Physical Problems	3.45 (2.21)	0.30	2.50 (2.04)	0.27	0.19–1.71	2.50	0.016
Brief COPE:							
Self-distraction	6.17 (1.50)	0.20	4.48 (1.46)	0.20	1.06–2.31	5.37	0.000
Active Coping	6.41 (1.46)	0.20	5.26 (1.42)	0.19	0.63–1.67	4.40	0.000
Denial	6.83 (1.55)	0.21	2.79 (1.25)	0.17	3.52–4.56	15.63	0.000
Substance use	5.20 (1.16)	0.16	2.11 (0.46)	0.06	2.75–3.43	18.21	0.000
Emotional support	4.25 (1.48)	0.20	2.82 (0.91)	0.12	−1.90–0.98	−6.28	0.000
Instrumental support	5.20 (1.38)	0.19	5.24 (1.17)	0.16	−0.47–0.39	−0.17	0.866
Behavioral disengagement	4.69 (1.49)	0.20	2.95 (1.15)	0.15	1.23–2.26	6.79	0.000
Venting	4.83 (1.30)	0.18	4.43 (1.19)	0.16	−0.06–0.88	1.73	0.090
Positive reframing	1.87 (0.88)	0.12	6.25 (1.53)	0.21	−4.87–3.90	−18.16	0.000
Planning	6.88 (1.24)	1.17	6.70 (1.56)	0.21	−0.37–0.73	0.65	0.520
Humor	4.30 (1.40)	0.19	4.20 (1.98)	0.26	−0.54–0.75	0.33	0.741
Acceptance	2.04 (0.27)	0.04	6.45 (1.37)	0.19	−4.80–4.04	−23.24	0.000
Religion	2.40 (0.66)	0.09	4.24 (1.72)	0.23	−2.27–1.40	−8.41	0.000
Self-blame	6.85 (1.55)	0.21	5.60 (1.42)	0.19	0.70–1.82	4.52	0.000

Note. GHQ-12 = General Health Questionnaire-12; DT = distress thermometer; M = means; SD = standard deviation; SE = standard error; Cl = confidence interval; LL = lower limit; UL = upper limit; GDL for *t* test = 56.

**Table 2:** Means, Standard Deviation, Standard Error and Confidence Interval for Scores on the GHQ-12, Distress Thermometer and Brief COPE. Participants in wellness courses.

Measure	Time 1		Time 2		95% Cl (LL–UL)	*t*	*p*
	M (SD)	SE	M (SD)	SE			
GHQ-12	5.22 (2.01)	0.29	3.35 (1.74)	0.25	1.22–2.54	5.74	0.000
DT:	5.61 (2.61)	0.45	3.97 (1.93)	0.34	0.52–2.75	2.98	0.005
Practical Problems	0.61 (0.87)	0.12	0.86 (1.02)	0.15	−2.17–0.17	−1.18	0.243
Family Problems	0.65 (0.75)	0.11	0.73 (0.73)	0.10	−0.38–0.22	−0.55	0.584
Emotional Problems	1.88 (1.27)	0.18	1.35 (0.97)	0.14	0.11–0.95	2.52	0.015
Spiritual/religious concerns	0.08 (0.28)	0.04	0.04 (0.20)	0.03	−0.04–0.12	1.00	0.322
Physical Problems	3.55 (2.58)	0.37	2.84 (2.62)	0.37	−0.37–1.80	1.32	0.192
Brief COPE:							
Self-distraction	4.50 (1.47)	0.21	5.94 (1.44)	0.21	0.91–1.97	5.47	0.000
Active Coping	6.92 (1.24)	0.18	5.08 (1.24)	0.18	1.31–2.36	7.03	0.000
Denial	6.96 (1.44)	0.21	2.72 (0.95)	0.14	3.71–4.75	16.38	0.000
Substance use	5.13 (1.26)	0.12	2.08 (0.39)	0.04	2.79–3.30	23.68	0.000
Emotional support	4.61 (1.66)	0.24	2.90 (0.29)	0.20	−1.9–1.20	−8.55	0.000
Instrumental support	4.94 (1.59)	0.23	5.06 (1.39)	0.20	−0.71–0.46	−0.43	0.671
Behavioral disengagement	4.53 (1.16)	0.12	2.91 (1.19)	0.14	1.23–1.98	8.47	0.000
Venting	4.89 (1.51)	0.22	4.63 (1.56)	0.23	−0.37–0.89	0.83	0.411
Positive reframing	1.82 (0.94)	0.09	6.08 (1.65)	0.11	−2.18–0.34	−0.16	0.876
Planning	6.82 (1.16)	1.16	6.85 (1.41)	0.14	−0.40–0.75	1.14	0.260
Humor	4.59 (1.68)	0.24	4.16 (1.97)	0.28	−0.27–1.12	1.24	0.222
Acceptance	2.15 (0.76)	0.08	6.59 (1.41)	0.14	−4.75–4.11	−27.25	0.000
Religion	2.51 (0.81)	0.08	4.29 (1.95)	0.19	0.99–2.43	−8.76	0.000
Self-blame	6.88 (1.53)	0.15	5.55 (1.61)	0.16	0.88–1.79	5.82	0.000

Note. GHQ-12 = General Health Questionnaire-12; DT = distress thermometer; M = means; SD = standard deviation; SE = standard error; Cl = confidence interval; LL = lower limit; UL = upper limit; GDL for *t* test = 56.
